# QuantiFERON-TB gold in-tube implementation for latent tuberculosis diagnosis in a public health clinic: a cost-effectiveness analysis

**DOI:** 10.1186/1471-2334-12-360

**Published:** 2012-12-19

**Authors:** Maunank Shah, Kathryn Miele, Howard Choi, Danielle DiPietro, Maria Martins-Evora, Vincent Marsiglia, Susan Dorman

**Affiliations:** 1 Johns Hopkins University School of Medicine, Baltimore, MD, USA; 2 Baltimore City Health Department, Baltimore, MD, USA; 3 Tulane University, New Orleans, LA, USA; 4 Johns Hopkins University, School of Medicine, Division of Infectious Disease, Center for TB Research, 1503 East Jefferson St, Room 118, Baltimore, MD, 21231, USA

**Keywords:** Tuberculosis, Diagnosis, Interferon-gamma release assay, Latent tuberculosis, Implementation

## Abstract

**Background:**

The tuberculin skin test (TST) has limitations for latent tuberculosis infection (LTBI) diagnosis in low-prevalence settings. Previously, all TST-positive individuals referred from the community to Baltimore City Health Department (BCHD) were offered LTBI treatment, after active TB was excluded. In 2010, BCHD introduced adjunctive QuantiFERON-TB Gold In-Tube (QFT-GIT) testing for TST-positive referrals. We evaluated costs and cost-effectiveness of this new diagnostic algorithm.

**Methods:**

A decision-analysis model compared the strategy of treating all TST-positive referrals versus only those with positive results on adjunctive QFT-GIT testing. Costs were collected at BCHD, and Incremental Cost-Effectiveness Ratios (ICERs) were utilized to report on cost-effectiveness.

**Results:**

QFT-GIT testing at BCHD cost $43.51 per test. Implementation of QFT-GIT testing was associated with an ICER of $1,202 per quality-adjusted life-year gained and was considered highly cost-effective. In sensitivity analysis, the QFT-GIT strategy became cost-saving if QFT-GIT sensitivity increased above 92% or if less than 3.5% of individuals with LTBI progress to active TB disease.

**Conclusions:**

LTBI screening with TST in low-prevalence settings may lead to overtreatment and increased expenditures. In this public health clinic, additional QFT-GIT testing of individuals referred for a positive TST was cost-effective.

## Background

Identifying and treating individuals with latent tuberculosis infection (LTBI) prevents progression to active TB disease and is a key component of tuberculosis (TB) control strategies in the United States (US) [1-3]. The US Centers for Disease Control and Prevention (CDC) currently recommends testing of only individuals at high risk for *M. tuberculosis* (MTB) infection or progression to active TB disease [3]. In the US, LTBI screening occurs through a mixture of public and private health sector efforts. In Baltimore City, initial screening for MTB infection is typically conducted by community providers, almost exclusively using the tuberculin skin test (TST) [4]. Baltimore City residents identified with a positive TST by community sources subsequently may be referred to the Baltimore City Health Department (BCHD), where they are provided, free of charge, with LTBI care. This care includes an assessment of active TB disease by symptom screening and chest x-ray, and LTBI treatment using CDC-recommended regimens once active TB disease is excluded. Prior to 2010, BCHD conducted no further LTBI testing for these referrals.

However, health department reliance on TST results from heterogeneous community sources has limitations [4]. Bacille Calmette-Guerin (BCG) vaccination in foreign-born individuals or exposure to non-tuberculous mycobacteria (NTM) may compromise TST specificity. Additionally, inter-reader variability of TST results may lead to misclassifications and false-positive results, particularly in low-prevalence populations [5]. These limitations may lower the positive predictive value of TST for identifying individuals at risk for progression to active TB disease [6].

Interferon-gamma release assays (IGRAs) are emerging tools for the detection of infection with *M. tuberculosis* with some advantages compared to TST, but are more costly. IGRAs, such as the QuantiFERON-TB Gold In-Tube (QFT-GIT, Cellestis, Ltd, Carnegie, Australia) test, are blood tests that measure *in vitro* interferon-gamma release from lymphocytes stimulated by antigens that are specific to *M. tuberculosis,* resulting in increased specificity and less cross-reactivity with NTM species or BCG-vaccination than TST [7-9]. QFT-GIT sensitivity is comparable to TST, and some studies suggest QFT-GIT may have a negative predictive value above 99% for identifying individuals at risk for progression to active TB disease [6,8,10]. The CDC has recommended that IGRAs may be used as an alternative to TST for LTBI testing in most circumstances, and may be preferred for individuals with a history of BCG-vaccination [9].

LTBI treatment can be resource-intensive for public health programs. In early 2010, BCHD implemented additional QFT-GIT testing as part of evaluations for individuals referred from community providers with suspected LTBI on the basis of a positive TST [4]. Subsequently, rates of LTBI diagnosis and treatment were substantially reduced with high rates of discordance between BCHD-directed QFT-GIT testing and TST results from the referral source; only 57% of foreign-born and 36% of US-born individuals referred for a positive TST had a positive QFT-GIT [4]. These findings are similar to those reported by others in low-prevalence settings. In a public health TB clinic in Alberta, Canada, QFT-GIT was used as a confirmatory test for patients with a positive TST and only 40% of patients were QFT-GIT positive [11]. Interpretation of discordance is challenging, but may represent low positive predictive value of TST for identifying LTBI in non-endemic settings due to suboptimal test specificity [11].

Adjunctive QFT-GIT testing of individuals referred to public health TB clinics for suspected LTBI on the basis of a positive TST will incur more diagnostic costs, but might reduce treatment of individuals with false-positive TSTs with consequent cost-savings; alternatively, it may also lead to missed LTBI diagnoses. This study sought to determine the costs associated with QFT-GIT implementation at BCHD, and to determine if adjunctive QFT-GIT testing was a cost-effective LTBI-care strategy for individuals referred on the basis of a positive TST, compared to a strategy of considering all TST-positive referrals to have true LTBI warranting treatment.

## Methods

This economic evaluation was conducted from a health-system perspective, with a target population consisting of individuals referred to public health clinics with suspected LTBI on the basis of a positive TST. Target audiences include city and state health departments, TB control programs, and public-sector healthcare payers. Individuals with close-contact to people with active TB disease were not considered as part of this analysis, since these represent a patient population with different LTBI risks. For the cost-effectiveness analyses, a one-year time-frame was used and the analytic horizon was extended to the life-expectancy of individuals with suspected LTBI; future costs and QALY’s were discounted at 3%. We additionally presented budgetary analysis using a 5 year time-horizon with and without discounting of future costs from the Public Health Department perspective. We conducted the analysis using TreeAge Software. This study did not involve human subjects; protocol was approved by ethics committees at the Johns Hopkins University School of Medicine (Baltimore, USA) and the Baltimore City Health Department.

### Study model

**Figure 1:**
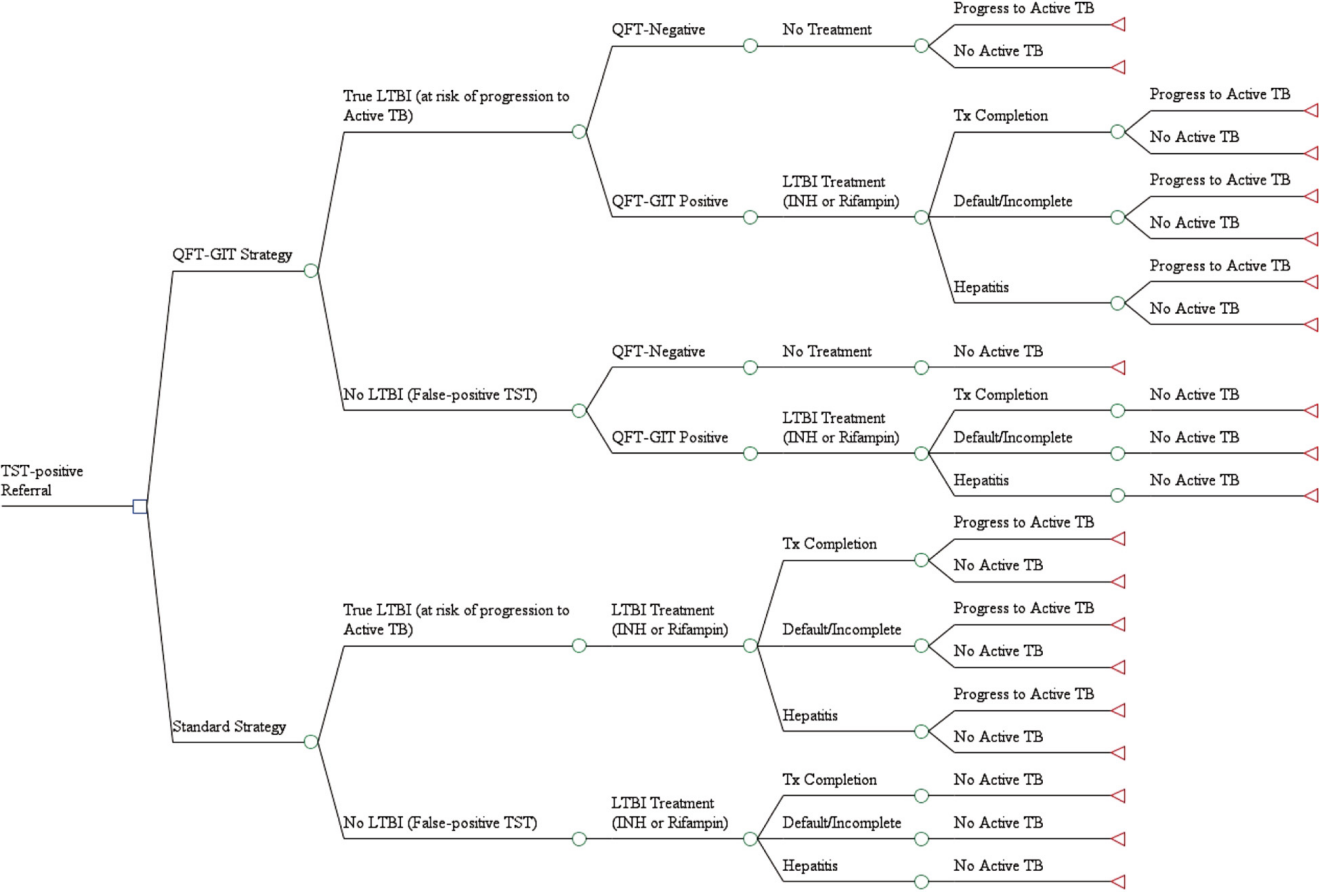
**Schematic of Decision Analysis Model for Cost-Effectiveness Evaluation.** Simplified schematic of decision-analysis model. Target population consists of TST-positive referrals to BCHD. LTBI prevalence among this population was estimated based on QFT-GIT positivity rates at BCHD. The model incorporates US and foreign-born to account for different LTBI prevalence in these two populations. In the Standard Algorithm, all individuals are offered LTBI treatment. In the QFT-GIT Algorithm, all individuals receive QFT-GIT and only those that are positive are provided LTBI treatment. LTBI treatment completion rates are based on current BCHD data. Individuals with incomplete treatment were considered to have only partial treatment efficacy. It is assumed that individuals without LTBI were unable to progress to active TB disease.

We compared two LTBI care strategies at BCHD for TST-positive referrals using a decision-analysis model (Figure 1): 

1) *Standard Algorithm (Standard)*: TST-positive individuals are referred to BCHD, which relies on referral-source TST results with no further LTBI diagnostic testing. All individuals are evaluated for active TB disease by symptom screen and chest x-ray, as well as additional testing as indicated. All individuals receive baseline liver chemistries. If active TB disease is excluded and there are no contraindications to LTBI treatment, then LTBI treatment is provided free-of-charge.

2) *QFT-GIT Intervention Algorithm (Intervention)*: TST-positive individuals are referred to BCHD, which subsequently conducts additional QFT-GIT testing on all referred individuals to assess LTBI status. All individuals are also evaluated for active TB disease by symptom screen and chest x-ray, as well as additional testing as indicated. All individuals receive baseline liver chemistries. QFT-GIT positive individuals, in whom active TB disease has been excluded and have no contraindications to LTBI treatment are provided LTBI treatment free-of-charge. Individuals with a negative QFT-GIT are not treated for LTBI.

### Key model parameters

Table 1 shows base-case values with ranges and sources for all key costs and model parameters. 

**Table 1:** Key Parameters for Cost-Effectiveness Analysis

**Variable**	**Base-Case**	**Low**	**High**	**Source**
**Epidemiologic/Diagnostic/Treatment Parameters**				
Lifetime LTBI progression to active TB	5%	1%	15%	[12,13]
Percent of LTBI referrals that are foreign-born	64%	30%	100%	[4]
Percent of QFT-GIT positivity in BCHD	57% FB; 36% US	--	--	BCHD, [4]
Prevalence of LTBI in TST-positive referrals	70% FB; 43% US	0%	100%	Calculated; [4]
Sensitivity of QFT-GIT	81%	36%	100%	[8,9,14]
Specificity of QFT-GIT	99%	90%	100%	[8]
Percent of LTBI patients treated with 4Rif	62%	0%	100%	BCHD
LTBI treatment completion for 9INH (4Rif)	52% (73%)	25%	100%	BCHD, [15,16]
Drug-induced liver injury (percent severe)	1% (0.002%)	0%	6%	BCHD, [13,15,17-20]
Efficacy of LTBI treatment medications	0.9	0.50	1	[3,17,21,22]
**QFT-GIT Costs**	2012 US$			
QFT-GIT tubes	$7.10/test	$6.60	$12.42	BCHD
Phlebotomy supplies and clinic supplies	$2.18	$0.55	$3.82	BCHD
QFT-GIT kit	$23.92/test*	$9.90*	$71.75*	BCHD
QFT-GIT lab consumables	$3.50/test	$.87	$6.12	BCHD
Total equipment costs for QFT-GIT testing††	$1.14/test	$.29	$1.99	BCHD
Phlebotomist labor for QFT-GIT	$1.60/test	$1.60	$3.99	BCHD
Laboratory labor for QFT-GIT	$2.28/test	$1.48	$2.52	BCHD
Overhead for QFT-GIT testing	$0.53/sample	$0.25	$2.50	BCHD
**TB Treatment Costs**				
Initial LTBI evaluation labor	$35.04	$15.88	$68.29	BCHD
Labor LTBI treatment: 9INH	$88.56	$36.40†	$171†	BCHD
Labor LTBI treatment: 4Rif	$44.28	$18.20†	$85.70†	BCHD
Drug costs 9INH	$26.82/course	$9.27	$91.35	BCHD
Drug costs 4Rif	$102.12/course	$102.12	$482.60	BCHD
Chest x-ray	$100	$50	$175	BCHD
Mild hepatitis	$41.62	$0	$344	BCHD, [21]
Severe hepatitis	$124.86	$41.62	$23,818	BCHD, [21]
Cost of active TB (includes drugs, staff, labs)	$8,568	$2,142	$64,195	BCHD, [20,21,23]
**Utilities**				
Well	1			[21]
9INH treatment	0.95	.9	.99	[20,21]
4Rif treatment	0.99	.9	.99	[20,21]
Mild hepatitis	0.85	1 mo	12 mo	[20,21]
Severe hepatitis	0.40	0.1	0.4	[20,21]
Active TB **	0.85	0.5	0.9	[21]
Fatal TB **	0.5	0.1	0.5	[21]
Death	0			[21]

*Note.* LTBI = latent tuberculosis infection; TB = tuberculosis; QFT-GIT = QuantiFERON-TB Gold In-Tube; TST = tuberculin skin test; 4Rif = 4-month regimen of Rifampin; 9INH = 9-month regimen of Isoniazid; BCHD = Baltimore City Health Department records.

* The base-case assumes a batch size of 24 patient samples per run. Low and high estimates are based on batch sizes ranging from 8 to 58 patient samples per run.

† Low and high estimates based on variable staff salary and time spent per appointment.

†† Includes costs for an Automated ELISA instrument, software, incubator, centrifuge, and other non-consumable laboratory equipment.

**Sensitivity analyses incorporated range of utility weights, along with low and high estimates for life-expectancy after TB disease, and low and high estimates of TB related mortality.

#### Epidemiologic, diagnostic, and treatment parameters

The incidence of active TB disease in Baltimore City in 2010 was 5.2/100,000 (population 620,961) [24]. Base-case analysis parameters were based on data from BCHD, which annually evaluates approximately 500 individuals, 64% foreign-born, referred by community providers for a positive TST [4]. Per routine care, all individuals referred for suspected LTBI are interviewed by clinical staff for demographics, medical history, and signs and symptoms of active TB disease, and also receive a chest x-ray and liver function tests. Patients with signs or symptoms of active TB disease are evaluated by sputum smear microscopy, culture, and other testing.

For the base-case analysis, 100% uptake of QFT-GIT testing and implementation was assumed with 3% of tests requiring repeat due to processing failure or an indeterminate result [4]. Without a true reference standard LTBI diagnostic test, it is impossible to ascertain the true proportion of LTBI among individuals referred to the BCHD with a positive TST; we assumed only patients with LTBI are at risk for progression to active TB disease. Among individuals referred to BCHD with a positive TST, only 58% of those who were foreign-born and 36% of those who were US-born had a positive QFT-GIT [4]. It is unknown if this test discordance represents false-positive TST results or false-negative QFT-GIT results, or some combination. Given these observed rates of QFT-GIT positivity, true LTBI prevalence among referrals was calculated based on estimated QFT-GIT sensitivity and specificity: true LTBI Prevalence = (% test positive + specificity −1)/(sensitivity + specificity −1). For the base-case, QFT-GIT sensitivity of 81% and specificity of 99% were assumed based on published literature [8,9,14]. Reported QFT-GIT sensitivity for LTBI may be underestimated since test performance has largely been assessed in people with active TB disease as a surrogate for LTBI. Longitudinal studies have shown that less than 1% of individuals with a negative QFT-GIT progress to active TB disease even in high-risk groups, suggesting higher QFT-GIT sensitivity for identifying LTBI [6,25,26]. Given parameter uncertainties, a wide range of QFT-GIT test characteristics and LTBI prevalence were explored in sensitivity-analysis.

At BCHD, individuals diagnosed with LTBI are treated with a 9-month regimen of Isoniazid (9INH) or a 4-month regimen of Rifampin (4Rif), as per CDC guidelines [3]. Treatment completion rates were incorporated into the model. For the base-case, lifetime risk of progression to active TB disease among those with untreated LTBI was assumed to be 5% at an average of 5 years from time of referral [6,12,21,25,27]. LTBI treatment regimens were assumed to have 90% treatment efficacy if completed [3,22]; given variability in timing of drug discontinuation and efficacy of partial treatment, incomplete treatment was assumed to offer on average 25% of complete treatment protection [17,21,22]. Individuals experiencing hepatotoxicity were assumed to complete only partial treatment. Mean age at time of referral to BCHD was 36 years (SD 16 years).

#### Outcome parameters

The primary outcomes were the expected costs per referral, Quality-Adjusted Life Years (QALYs) accrued per referral, and expected active TB disease cases per referral comparing the *Intervention* to *Standard* algorithms. Cost-effectiveness was represented using Incremental Cost-Effectiveness Ratios (ICERs), expressed as $US Dollars/QALY-gained [28]; a probabilistic sensitivity analysis was additionally performed using monte-carlo simulation methods to generate 95% confidence intervals and explore varying willingness to pay thresholds. QALYs accrued based on years of remaining life experienced with utility weights shown in Table 1[29]. Mortality for individuals developing active TB disease was assumed to be 5% in the base-case [21,30]. Future QALYs were discounted at 3%.

#### Costs

Costs associated with LTBI evaluation and treatment were collected at BCHD. QFT-GIT test costs were collected from the time of specimen collection through reporting of completed test results. QFT-GIT is currently performed at an off-site local BCHD laboratory at a location separate from the BCHD TB clinic, with samples transported daily. An “ingredients” approach was used, which involved multiplying input quantities used by unit prices. Staff time, consumable supplies, and equipment quantities utilized for performing the QFT-GIT test were determined through direct observation of testing procedures and time-motion studies. QFT-GIT tests were assumed to be performed in batches of 24 based on current laboratory practice and batch size was varied in sensitivity-analysis. Capitol item costs were estimated from manufacturer quotations and/or laboratory invoices and were annualized over their useful lifespans as estimated by the laboratory manager. Overhead laboratory costs were determined, including those for quality assurance, specimen transport, supply delivery, and estimates for rent and utilities attributable to QFT-GIT testing based on building space and volume of testing. Costs of LTBI treatment included drug costs based on invoices. Labor costs were determined based on clerical and clinician staff time spent during an average office visit. One office visit was included for initial evaluation and monthly office visits were included for patients on LTBI treatment. Translation service costs were added for non-English speaking patients. Costs associated with drug toxicity were based on BCHD estimates of additional diagnostic testing and clinic visits. There is little recent published literature on the downstream costs associated with developing active TB disease [21,23]. As such, base-case estimates for BCHD costs per patient were based on current staff, drug, and diagnostic expenditures for an average active TB case; uncomplicated active TB cases were assumed to require only outpatient treatment with no hospital days. Each individual was assumed to receive standard drug treatment, directly observed, with routine monitoring for treatment response and toxicity [31]. Potential inpatient hospitalization costs associated with active TB disease were incorporated into the sensitivity-analysis. All key costs were explored in sensitivity-analysis; when published estimates of cost ranges were unavailable, potential price variations of 75% were explored. Costs are presented in $US 2012 and future costs were discounted at 3%.

## Results

### QFT-GIT testing costs

In the base-case, the total cost of performing the QFT-GIT test at BCHD was $43.51 per test; of total test costs, QFT-GIT tubes accounted for 17% ($7.10 per test), the QFT-GIT ELISA kit for 57% ($23.92 per test), and labor 9% ($3.88 per test), with the remaining 17% ($7.34 per test) attributable to other supplies, equipment, and overhead (Table 1). If batch size of QFT-GIT tests per run were increased from the BCHD average of 24 to 58 as per manufacturer maximum, net QFT-GIT cost was $27.68 per test; net QFT-GIT cost was $97.47 for a batch size of 8. In sensitivity-analyses, net QFT-GIT cost per individual ranged from $22.17 to $108.25 when component costs for consumables, overhead, labor, and equipment were varied.

### Cost of LTBI-care for TST-positive referrals

In the base-case, the *Intervention* cost an additional $10.12 per referral compared to the *Standard* ($370.04 versus $359.93) when all health-system costs were considered (Table 2). When examining only LTBI-care costs (i.e. excluding downstream active TB disease costs), the *Intervention* saves $22.89 per referral compared to the *Standard* ($252.50 versus $275.39, Table 2), with total LTBI-related program savings of $11,445 per year (assuming 500 referrals per year, Table 3). These LTBI savings were offset by potential additional costs ($33.00 per referral, Tables 2 and 3) related to more predicted active TB cases in the *Intervention* compared to *Standard* when all health-system costs are considered. Assuming QFT-GIT sensitivity was 100% for identifying LTBI patients, the *Intervention* would be less costly compared to the *Standard* ($353.29 versus $359.93). 

**Table 2:** Costs and Effects of Intervention Compared to Standard Algorithm in the Base-Case

**Variable**	**Standard Strategy**	**QFT-GIT Strategy**	**Incremental**
**Costs**			
*Costs per Individual*			
QFT-GIT testing costs	$0.00	$43.51	$43.51
LTBI treatment and monitoring	$275.39	$208.99	-$66.40
Total LTBI-care costs	$275.39	$252.50	-$22.89
Active TB costs per individual*	$84.54	$117.54	$33.00
Net costs per individual	$359.93	$370.04	$10.12
**Effects**			
QALYs**	25.21 per referral	25.22 per referral	0.01 QALYs gained per referral
Active TB	9.9 per 1000 referrals	13.7 per 1000 referrals	3.8 per 1000 referrals
**Cost-Effectiveness**			
*Base-Case*	--	--	$1,202 per QALY-gained†

*Note.* QFT-GIT = QuantiFERON-TB Gold In-Tube; LTBI = latent tuberculosis infection; TB = tuberculosis; BCHD = Baltimore City Health Department; QALYs = Quality-Adjusted Life Years.

*Active TB costs attributable to individuals referred to the BCHD TB Control Program who go on to develop active TB disease later in life; future costs were discounted at 3%.

**Future QALY’s were discounted at 3%.

†ICER calculated as incremental costs divided by incremental effects ($10.12/0.01QALY’s gained).

**Table 3:** Health Department Costs and Budgetary Impact Per Year (500 Referrals)

**BCHD TB Control Program Costs**	**Standard Strategy**	**QFT-GIT Strategy**	**Incremental**
Referrals per year	500	500	
Total QFT-GIT testing costs per year	$0.00	$21755.00	$21755.00
Total LTBI treatment and monitoring costs per year	$137695.00	$104495.00	-$33200.00
Total LTBI-care costs per year	$137695.00	$126250.00	-$11445.00
Total active TB costs per year*	$42270.00	$58770.00	$16500.00
Net costs	$179965.00	$185020.00	$5060.00

*Active TB costs attributable to individuals referred to the BCHD TB Control Program who go on to develop active TB disease later in life using a 5 year analytic time horizon; future costs were discounted at 3%. Without discounting, the costs would be $49,005 and $68,135 for the Standard and QFT-GIT strategies, respectively.

There were several scenarios under which the *Intervention* was less costly compared with the *Standard*, including when QFT-GIT test costs are below $33.40, 4RIF drug costs increase above $142 per treatment course, or QFT-GIT sensitivity is above 93%. However, incremental costs for the *Intervention* compared to the *Standard* were greater with rising active TB disease-associated costs, increased progression rates to active TB disease, or higher QFT-GIT testing costs.

### Impact on QALYs and progression to active TB

In the base-case, the *Intervention* was more effective than the *Standard* (0.01 QALYs-gained per referral for *Intervention*). This benefit was seen despite a slightly higher expected rate of progression to active TB disease with the *Intervention* in the base-case (incremental increase of 3.8 active TB disease cases per 1000 referrals). In one-way sensitivity-analysis, the incremental benefit associated with the *Intervention* was most sensitive to the treatment regimen used for LTBI and increased to 0.022 QALYs gained per referral if 9INH is preferentially used over 4Rif. The only condition in which the *Standard* was considered more effective was if lifetime progression rates to active TB disease among those with LTBI increase above 14%.

### Cost-effectiveness

For the base-case scenario, implementation of QFT-GIT for adjunctive testing was associated with an ICER of $1,202 per QALY-gained compared to the *Standard*, and would be considered highly cost-effective compared to a willingness-to-pay (WTP) threshold of $50,000/QALY-gained for the US [32]. Without 3% annual discounting, the ICER rises to $3,331 per QALY-gained.

**Figure 2:**
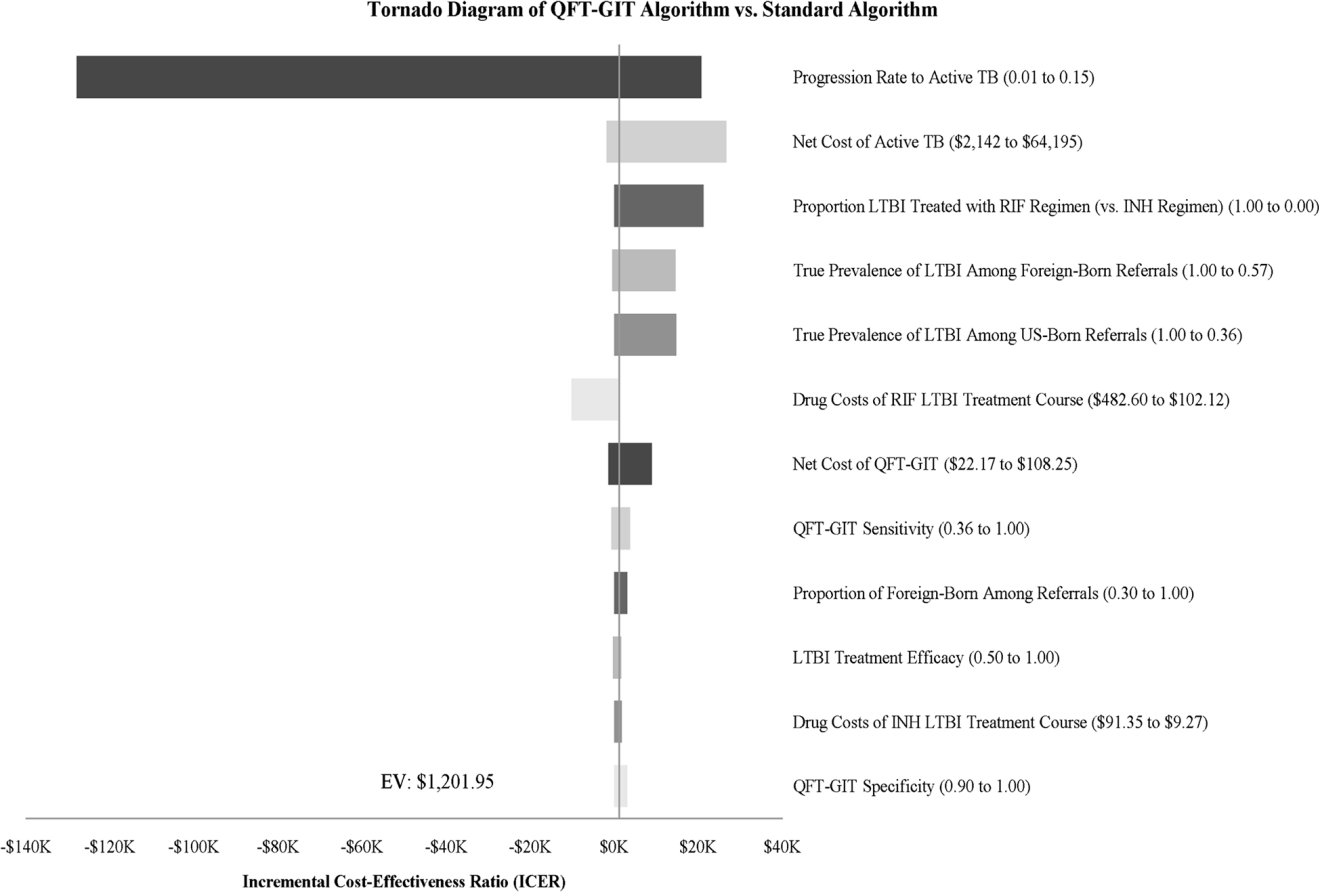
**One-Way Sensitivity-Analysis of Key Model Parameters**. Line represents incremental cost-effectiveness ratio (ICER) when using base-case estimates of all parameters. Not all parameters tested in sensitivity-analysis are shown. Top 12 factors affecting ICER are shown. EV-expected value of the ICER under base-case parameters.

One-way sensitivity-analyses were performed on all key model parameters, and Figure 2 shows the variables found to have the most effect on the base-case ICER. There were no conditions in which the ICER for the *Intervention* rose above the WTP threshold, and the *Intervention* was therefore considered the preferred algorithm. There were several conditions in which the *Intervention* would be considered cost-saving. In particular, the *Intervention* dominates (i.e. cheaper and more effective) the *Standard* algorithm when QFT-GIT sensitivity is above 92%, 4RIF drug costs increase above $142 per treatment course, or if less than 2.5% of individuals with LTBI progressed to active TB disease over their lifetime. 

We additionally explored the impact of assumptions regarding utility weights of LTBI therapy and active TB disease. If QALY losses attributable to LTBI therapy with INH are reduced (i.e. utility weight increased to 0.99), the ICER comparing the *Intervention* to *Standard* increases to $14452 per QALY-gained. The ICER ranged from $1180-$1400 per QALY gained when the utility weight associated with active TB disease was varied between 0.9 and 0.5; if mortality among those developing TB disease increases above 14%, the *Standard* algorithm was found to dominate the *Intervention.* Drug toxicity was found to have little impact on the ICER ($999 to $1246 per QALY-gained, when percentage experiencing LTBI treatment related liver injury was varied from 0-6%, respectively).

**Figure 3:**
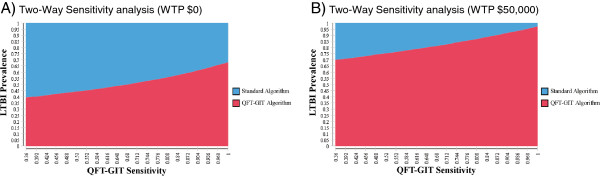
**Two-Way Sensitivity-Analysis of QFT-GIT Sensitivity and LTBI Prevalence.** Blue represents parameters at which Standard Algorithm is preferred option and red represents parameters at which QFT-GIT Algorithm is the preferred option given willingness to pay threshold (WTP): **A**) Two-way sensitivity analysis of LTBI prevalence versus QFT-GIT sensitivity at WTP threshold of $0 per QALY-gained; **B**) Two-way sensitivity analysis of LTBI prevalence versus QFT-GIT sensitivity at WTP threshold of $50,000 per QALY-gained.

Two-way sensitivity-analyses further examined the impact of LTBI prevalence among TST-positive referrals and QFT-GIT sensitivity (Figure 3). At current WTP threshold, the QFT-GIT testing strategy remained the preferred option at most permutations of LTBI prevalence and QFT-GIT sensitivity, and became cost-saving regardless of QFT-GIT sensitivity if true LTBI prevalence among TST-positive referrals fell below approximately 40%. For scenarios of true LTBI prevalence greater than 95% among TST-positive referrals, treating all individuals becomes the preferred strategy regardless of QFT-GIT sensitivity. 

**Figure 4:**
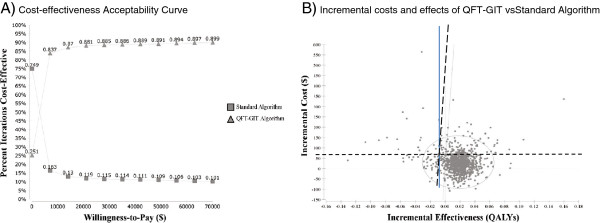
**Results from a Probabalistic Sensitivity-Analysis using Monte Carlo Simulation Methods.A**) Cost-effectiveness acceptability curve showing probability that the intervention will be cost-effective compared to treating all TST-positive referrals at varying willingness-to-pay thresholds. **B**) Incremental cost-effectiveness of QFT-GIT vs. Standard Algorithm during iterations of Monte Carlo Simulation. Ellipse represents 95% confidence points. Diagonal dashed line represents ICERs at a WTP threshold of $50,000. Points to the right of this dashed line are considered cost-effective. Dotted horizontal line shows incremental cost of $0.

Monte-Carlo simulation methods were used to conduct a probabilistic sensitivity-analysis (PSA) and results are shown in Figure 4. The mean ICER was $2,408 per QALY-gained [95%CI; -$12,480 to $21,496]. The mean incremental cost for the *Intervention* was $30.53 per referral [95%CI; -$54.68 to $143.79]; the mean incremental effect was 0.018 QALYs-gained [95%CI; -0.02 to 0.05]. The *Intervention* was considered cost-effective 89.1% of the time (WTP $50,000) and the *Intervention* dominated the *Standard* 25% of the time (i.e. WTP $0). 

## Discussion

In the US, LTBI screening and treatment occurs in the public and private health sectors with costs spread amongst different entities. In Baltimore City, LTBI screening is largely conducted by community sources using TST, while treatment is typically provided by BCHD [4]. LTBI treatment of all TST-positive individuals, however, is costly and labor intensive and may lead to overtreatment in this low prevalence setting [4,5,11]. These results suggest that BCHD implementation of adjunctive QFT-GIT testing for individuals referred by community sources with a positive TST is highly cost-effective and potentially cost-saving.

Previously, there has been limited cost data to guide QFT-GIT implementation in US public health clinics [21,33,34]. As currently implemented at BCHD, the QFT-GIT test costs $43.51 per individual when all test-related factors were considered and were strongly influenced by testing volume.

The optimal algorithm for LTBI care is likely to be impacted by TB program structure, resources, outcome preferences, and regional epidemiology. This analysis provides several details to aid programmatic decision-making with respect to QFT-GIT use. For example, at BCHD, staff available for LTBI care is limited. Providing LTBI care and treatment for all TST-positive referrals (at a cost of $275 per individual) resulted in annual LTBI associated program costs exceeding $137,000. BCHD implementation of adjunctive QFT-GIT testing in 2010 substantially reduced the number of individuals diagnosed with and treated for LTBI and is expected to save the program nearly $23 per referral in LTBI-related costs [4]. Such savings may allow public health departments to reallocate LTBI resources to other activities, including care of active TB patients and their contacts.

On the other hand, this study also suggests that adjunctive QFT-GIT testing for TST-positive individuals may result in a small proportion of individuals with true *M. tuberculosis* infection remaining untreated, resulting in nearly four additional cases of active TB disease per 1,000 referrals. When active TB disease-associated costs were considered, QFT-GIT implementation at BCHD resulted in a net increase in health-system costs of $10.12 per referral compared to treating all TST-positive individuals. Importantly, however, the QFT-GIT testing strategy was associated with overall increases in QALYs despite the small increase in active TB cases, a consequence of substantially fewer individuals being prescribed LTBI treatment and less related adverse events. This analysis highlights the need for programs to carefully weigh the potential health benefits of reduced LTBI treatment with the potential risk of slight increases in active TB cases. Moreover, sensitivity-analysis demonstrated that QFT-GIT implementation became the dominant strategy (both cost-saving and more effective) if true QFT-GIT sensitivity for detecting LTBI is above 92% or if the proportion of individuals with LTBI progressing to active TB disease is less than 3.5%.

This study has several limitations. Conducting economic evaluations of LTBI diagnostic algorithms is complicated by uncertainty about true LTBI prevalence among those with discordant TST and QFT-GIT results. Determining whether an individual has true LTBI is challenging given the lack of a reference standard test, making sensitivity and specificity estimates for diagnostic tests less certain. Moreover, the target population consisted of individuals referred from heterogeneous community sources in whom there is likely a broad range of true *M. tuberculosis* infection risk and of progression to active TB disease. Nonetheless, extensive sensitivity-analyses explored the impact of variability in all key parameters; QFT-GIT implementation was cost-effective nearly 90% of the time at current WTP thresholds. This analysis also assumed complete reliance on QFT-GIT test results to determine LTBI status. Per current guidelines, clinicians may incorporate information about quantitative TST and QFT-GIT results, immunosuppression, and other clinical information to determine LTBI status [4,9]. Finally, this study was limited to assessing cost-effectiveness of QFT-GIT testing for a population of TST-positive individuals in a low prevalence setting and our results may not apply to all other risk groups or settings.

## Conclusions

Overall, this study offers detailed cost information for both QFT-GIT test implementation and LTBI treatment in a representative public health TB program in the US. Given reductions in public health funding, optimizing resources for TB control is critical and this analysis suggests that incorporation of QFT-GIT testing for TST-positive individuals is likely highly cost-effective in low-prevalence settings. Further studies are needed to assess rates of progression from LTBI to active TB disease in individuals with discordant TST and QFT-GIT test results. Recent literature suggests that QFT-GIT may have a high negative predictive value for identifying those at risk for progression to active TB disease [6,25,26]; in this scenario, a strategy of adjunctive QFT-GIT testing is likely to be not only cost-effective but cost-saving.

## Competing interests

The authors declare that we have no competing interests.

## Authors’ contributions

MS performed the data collection, analyzed the data, led the manuscript writing. KM analyzed the data and assisted with manuscript preparation. HC analyzed the data and assisted with manuscript preparation. DD collected data and assisted with data analysis. MM conceived the study and assisted in study design. VM performed data collection, assisted in study design, and provided technical expertise. SD conceived and supervised the study. All authors read and approved the final manuscript.

## Pre-publication history

The pre-publication history for this paper can be accessed here:

http://www.biomedcentral.com/1471-2334/12/360/prepub
